# A Letter to the Editor regarding the article “gastric epithelial neoplasm of fundic-gland mucosa lineage: representative of the low atypia differentiated gastric tumour and Ki67 may help in their identification”

**DOI:** 10.3389/pore.2026.1612588

**Published:** 2026-09-30

**Authors:** Houqiang Li, Lanqing Zheng, Guodong Zhong, Linying Chen, Xin Chen

**Affiliations:** 1 Department of Pathology, Fuzhou University Affiliated Provincial Hospital, Fujian Provincial Hospital, Fuzhou, China; 2 Shengli Clinical Medical College of Fujian Medical University, Fuzhou, China; 3 The Second Affiliated Hospital of Fujian Traditional Chinese Medical University, Fuzhou, China; 4 The First Affiliated Hospital of Fujian Medical University, Fuzhou, China

**Keywords:** AI, biopsy, gastric tumour, Ki67, oxyntic gland adenoma

We have recently published a study entitled “Gastric epithelial neoplasm of fundic-gland mucosa lineage: representative of the low atypia differentiated gastric tumour and Ki67 may help in their identification” in *Pathology & Oncology Researc*h [1]. In the aforementioned study, the clinicopathological characteristics of 37 gastric epithelial neoplasms of fundic-gland mucosa lineages (GEN-FGMLs) were characterised, and it was proposed that a Ki67 proliferation index greater than 2.5% and a lesion size greater than 4.5 mm could assist in differentiating oxyntic gland adenoma (OGA) from gastric adenocarcinoma of the fundic-gland type (GA-FG) and fundic-gland mucosa type (GA-FGM). Subsequently, these findings were presented at the 37th European Congress of Pathology (Vienna, 6–10 September 2025), with the abstract published in Virchows Archiv [2]. In this study, we aim to provide a comprehensive elaboration on the clinical diagnostic pathway that integrates these quantitative biomarkers. This approach is designed to address the prevailing challenges in biopsy interpretation, thereby facilitating more precise and informed clinical decisions.

In the multicenter cohort of 47 GEN-FGML cases (24 OGAs, 21 GA-FGs, and 2 GA-FGMs), it was confirmed that GA-FG/GA-FGM lesions were significantly larger than OGAs (p < 0.0001) and exhibited markedly higher Ki67 proliferation indices (median 7.5%, range 2%–26% vs. median 1.9%, range 1%–5% for OGA; p < 0.0001). ROC analysis validated the Ki67 cutoff of 2.5% (AUC = 0.9476, 90% sensitivity, 90.48% specificity) and the size threshold of 4.5 mm (94.74% sensitivity, 92.37% specificity) proposed in our original study (Figure 1A). Decision Curve Analysis (DCA) confirmed that Ki67 offered superior net benefit in moderate-risk ranges (up to 0.9 at 0.3–0.6 threshold probabilities), while the size model performed best at lower risk thresholds (0.8 at 0.2–0.4) (Figure 1B). These findings lend support to a dual-parameter diagnostic workflow, but the key to clinical translation lies in standardising Ki67 assessment.

**FIGURE 1:**
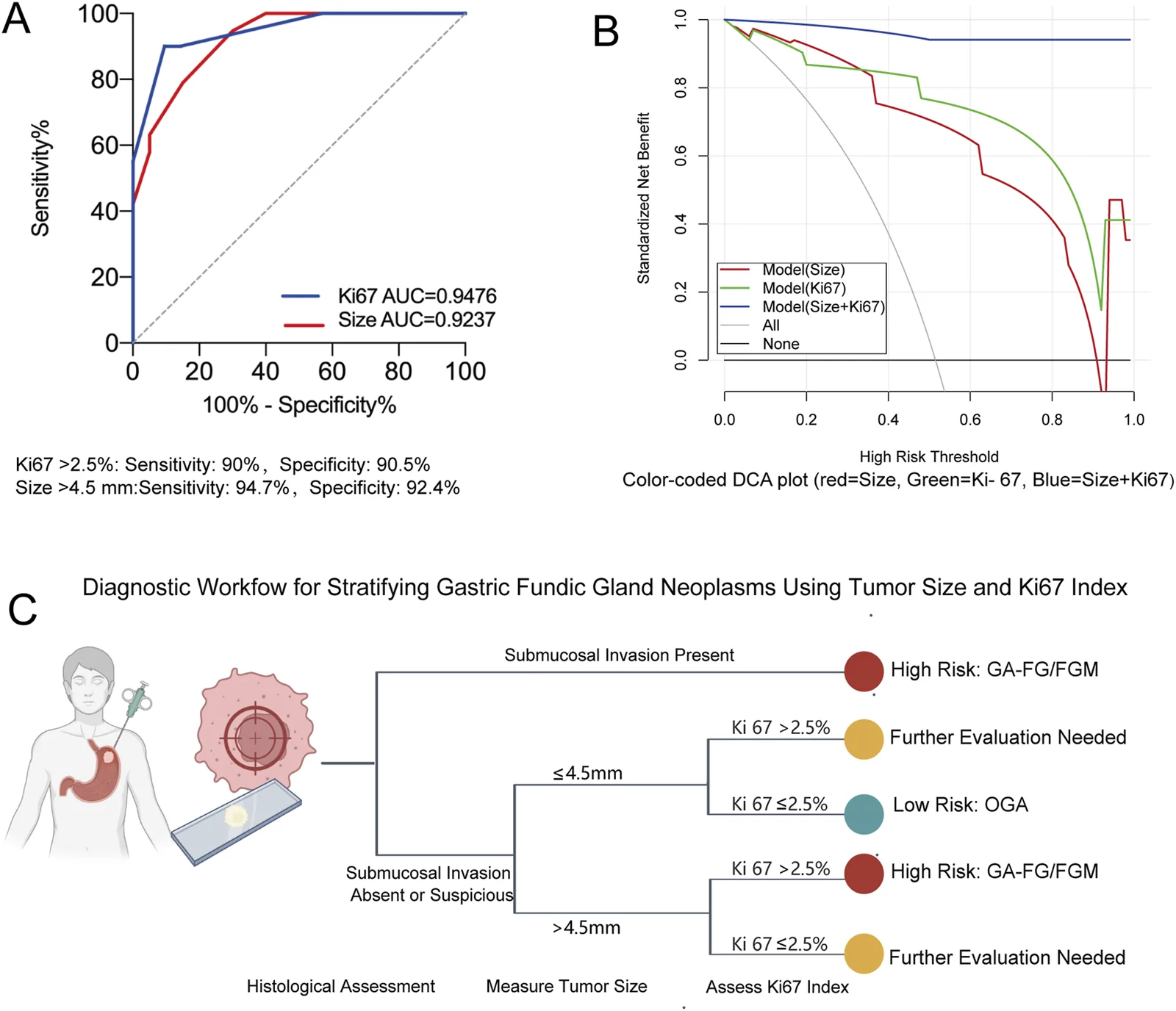
**(A)** ROC curves for Ki-67 index and tumor size in differentiating OGA from GA-FG/GA-FGM, with optimal cutoffs of 2.5% (AUC = 0.9476) and 4.5 mm (AUC = 0.9237), respectively. **(B)** Decision curve analysis (DCA) demonstrating the net clinical benefit of the Ki-67 model (peak net benefit 0.9 at threshold probabilities 0.3–0.6) and the size model (peak 0.8 at 0.2–0.4). **(C)** Proposed diagnostic workflow integrating tumor size measurement and Ki-67 index to classify GEN-FGMLs into OGA (low risk) versus GA-FG/GA-FGM (high risk), guiding biopsy interpretation and treatment decisions.

Ki67 quantification is notoriously subject to inter-observer variability, especially in small biopsy specimens with limited tumour area. To address this challenge, an AI-based nucleus recognition algorithm (Domain-Specific Pruning) was employed, which was specifically trained on Ki67-immunostained slides from GEN-FGMLs. This algorithm demonstrated high concordance with manual scoring by pathologists, while also enhancing reproducibility [3]. In the proposed diagnostic pathway (Figure 1C), the initial step involves the measurement of macroscopic size, which can be readily obtained from endoscopic images or resection specimens. In the event of a lesion measuring ≤4.5 mm and exhibiting a Ki67 index ≤2.5%, the lesion can be classified as OGA with a high degree of confidence (specificity >90%), thus permitting conservative management or further observation without the necessity for immediate endoscopic resection. Conversely, if either parameter exceeds its threshold, the lesion should be considered for endoscopic resection, as it is likely to be GA-FG or GA-FGM. In instances where size and Ki67 results are discordant (e.g., small size but high Ki67, or large size but low Ki67), we advocate proceeding with resection due to the enhanced sensitivity of size (94.7%) and the superior specificity of Ki67 (90.5%), which collectively minimise both missed diagnoses and unnecessary interventions.

The workflow in question offers several advantages: The provision of objective, quantitative criteria is of particular value in the context of biopsy samples, where submucosal invasion cannot be assessed. Secondly, it serves to reduce inter-pathologist variability, a recognised source of diagnostic inconsistency in these rare tumours. Thirdly, it is readily implementable in routine practice, as both size measurement and Ki67 immunohistochemistry are universally available. It is acknowledged that prospective validation in larger, independent cohorts, particularly across different ethnic populations, is still required. However, the findings of this study indicate that a combination of macroscopic size and Ki67 levels may offer a pragmatic and high-accurate diagnostic framework that can guide clinical decision-making and prevent overtreatment of indolent OGAs.

In summary, the multicentre validation study corroborates the diagnostic value of the Ki67 and size criteria that were originally proposed. The integration of Ki67 quantification into a simple dual-parameter pathway holds promise for standardising the diagnosis of GEN-FGMLs, especially in biopsy settings. It is hoped that this approach will stimulate further studies to optimise management strategies for these unique gastric neoplasms.

## Data Availability

The raw data supporting the conclusions of this article will be made available by the authors, without undue reservation.
